# Supplementary material for: Influence of Lipid Composition of Cationic Liposomes 2X3-DOPE on mRNA Delivery into Eukaryotic Cells
Source: Pharmaceutics. 2022 Dec 20;15(1):8. doi: 10.3390/pharmaceutics15010008 (PMC9860636; doi:10.3390/pharmaceutics15010008)
Supplement: Supplementary file 1 [file pharmaceutics-15-00008-s001.zip › pharmaceutics-2074650-supplementary.pdf]

pharmaceutics

MDPI

Supplementary materials

S1. Characterization of 2X3 by NMR

2X3: beige solid, mass-spectra: found *m/z* 1226.3 [M – 4 HCl + H]$^+$, calculated for $C_{78}H_{144}Cl_4N_6O_4$: 1225.13 [M – 4 HCl]$^+$. NMR ^{1}H (DMSO-d$_6$ - $CDCl_3$, 1 : 3 vol.): 0.61 (s, 6 H, 2 C(13)Me), 0.79 (d, 6 H, *J* = 6.5, 2 C(25)Me), 0.80 (d, 6 H, *J* = 6.5, 2 C(25)Me), 0.83 (d, 6 H, *J* = 6.5, 2 C(20)Me), 0.94 (s, 6 H, 2 C(10)Me), 0.91-2.04 (m, 76 H, protons Chol, $NHCH_2(C\underline{H}_2)_2CH_2NH$, 2 $NCH_2C\underline{H}_2CH_2N$, 2 $CH_2(C\underline{H}_2)_4CH_2$), 2.12-2.35 (m, 4 H, 2 $H_2C(4)$ Chol), 2.40-2.61 (m, 8 H, 2 $C\underline{H}_2NHC\underline{H}_2$), 2.99-3.27 (m, 12 H, 2 $C\underline{H}_2NH$, 4 NCH_2), 4.34-4.49 (m, 2 H, 2 H(3) Chol), 4.57-4.70 (m, 2 H, 2 NH), 5.26-5.33 (m, 2 H, 2 H(6) Chol).

S2. Quality assessment of mRNAs after in vitro transcription

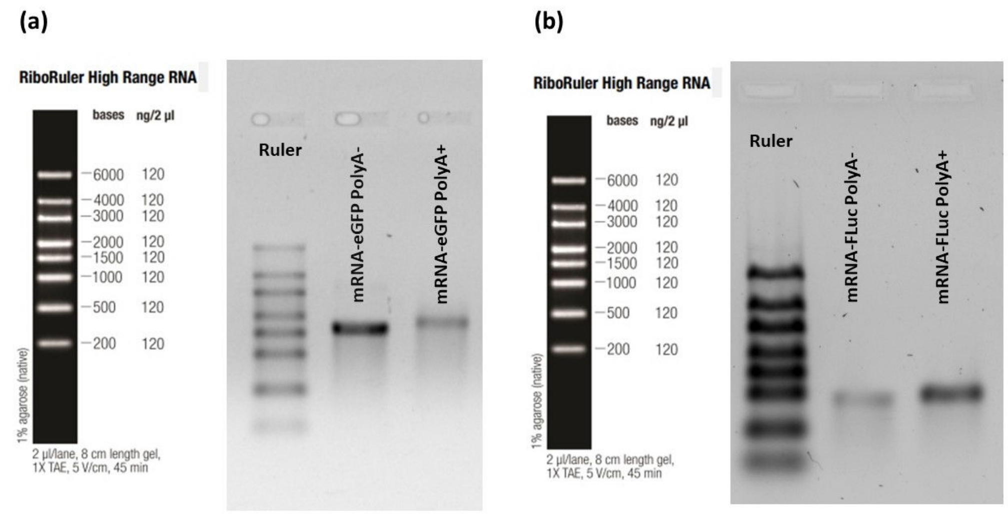

Figure S1. Quality assessment of mRNAs after in vitro transcription by using 1% (w/v) agarose gel in 1× TBE buffer under denaturing conditions. (a) mRNAs encoding eGFP (mRNA-eGFP): Thermo Scientific RiboRulerHigh Range RNALadder #SM1821, mRNA-eGFP PolyA-, mRNA-eGFP PolyA+. (b) mRNAs encoding firefly luciferase (mRNA-FLuc): Thermo Scientific RiboRulerHigh Range RNALadder #SM1821, mRNA-FLuc PolyA- , mRNA-FLuc PolyA+.

S3. Cytotoxicity studies

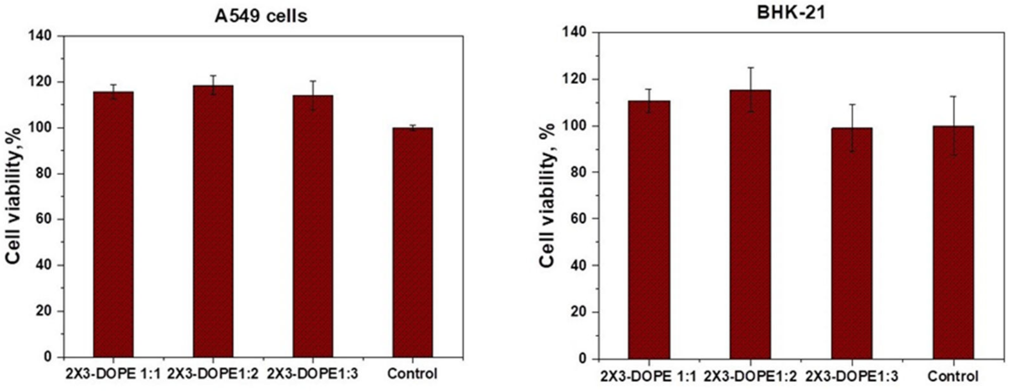

Figure S2. Cell viability of A549 and BHK-21 cells at 24 hours after the treatment with the cationic liposomes. Error bars represent ± SD (n=3).
